# Supplementary material for: Feasibility of machine learning analysis for the identification of patients with possible primary ciliary dyskinesia
Source: Orphanet J Rare Dis. 2025 Oct 14;20:516. doi: 10.1186/s13023-025-03966-z (PMC12522419; doi:10.1186/s13023-025-03966-z)
Supplement: Supplementary file 2 — Supplementary Material 2. [file 13023_2025_3966_MOESM2_ESM.docx]

**e-Appendix-2: ICD10 / CPT / Drug Coding Reference for Primary Ciliary Dyskinesia**

[**INTRODUCTION 3**](#_hhgjr2ei9qym)

[**DIAGNOSIS CATEGORIES AND MEASURES 3**](#_tdugq41n34tm)

[**ICD DIAGNOSIS CODES 5**](#_m4jrxh7ra9ku)

[AIRWAY - Atelectasis 5](#_13zf2kbe19oz)

[AIRWAY - Breathing Issues 5](#_hmpnxvisbfsw)

[AIRWAY - Bronchiectasis 5](#_wikxbyeawksq)

[AIRWAY - Bronchitis 5](#_jd7usqbcbg8s)

[AIRWAY - Cough 6](#_2kh2ufan2wp)

[AIRWAY - Serious Pulmonology Events 6](#_ha043ikp1br4)

[AIRWAY - Pneumonia 7](#_n6qj1et55qy4)

[AIRWAY - Upper Airway Infections 7](#_i8irfeevqhkk)

[CM - Congenital Malformations of Heart 7](#_7f9my9py059m)

[CM - Other Congenital Malformations 8](#_hlm3bwdr5q0k)

[CM - Situs Inversus 8](#_k2bp5tomdfkm)

[DIAG. - Asthma 8](#_jl639curgyph)

[DIAG. - COVID-19 8](#_obvu4z42cdo7)

[DIAG. - Cystic Fibrosis 9](#_euhwmtipb3c8)

[DIAG. - Pulmonary Diagnoses 9](#_dtzpyau065ic)

[DIAG. - Q348 - Congenital Malf. Resp. Sys. (Specific) 9](#_5mldmn9st1ik)

[EAR - Otitis media 9](#_i9cjlt2ysv4a)

[EAR - Hearing Loss 10](#_argm0nxpzp3d)

[EAR - Otorrhea 12](#_oza8iuqnhrze)

[FERTILITY - Infertility 12](#_9jq93wlso31z)

[IMMUNE SYSTEM - Immunodeficiency 12](#_a3h9vhcts2qu)

[NOSE - Nasal Congestion 13](#_c01j3inds1cm)

[NOSE - Nasal Polyps 13](#_ddcggcru3cy)

[NOSE - Rhinitis 13](#_3mvylkxf8khu)

[QOL - Depression or Anxiety 13](#_pj93wet6l5nz)

[SINUS - Chronic Sinusitis 14](#_i09rnrk9nqux)

[**PROCEDURE CATEGORIES 14**](#_bfh9l5sfsq0g)

[**PROCEDURE CODES 15**](#_gfj749ocbm0i)

[PROC. - Acapella/Flutter 15](#_a13ebhj3d7i)

[PROC. - Adenoidectomy 15](#_a4w1wusv6t40)

[PROC. - BAHA 15](#_qhgnm3rwtmu5)

[PROC. - Bronchoalveoloar lavage 15](#_89fx6ks2y3q)

[PROC. - Cochlear Implant Placement 17](#_5mgtn4p8hc3z)

[PROC. - Cochlear Implant Maintenance 18](#_dbepv2v1h2v0)

[PROC. - Chest Wall Manipulation 19](#_9fop8w8t7l8o)

[PROC. - Chest X-Rays 19](#_9u1x1te8f440)

[PROC. - Diagnostic EM 19](#_q3nink31w09o)

[PROC. - Endoscopic airway exam 19](#_70a6nouj6y5k)

[PROC. - Genetic Testing 19](#_y7xs501xglva)

[PROC. - Hearing aid Fitting + Maintenance 20](#_usbyiy5s714a)

[PROC. - Hospitalization 22](#_zifdt3qf569g)

[PROC. - IPV 22](#_q6agr5xgfgxa)

[PROC. - Lung Function Measurement 22](#_vvmqkktoict8)

[PROC. - Lung Transplant 23](#_vfwkfyer6klf)

[PROC. - Mastoidectomy 23](#_6rjz10ti05rj)

[PROC. - Myringotomy Tubes 24](#_9a8mq2oujypt)

[PROC. - Nasal Biopsy 24](#_yeday24ahtdr)

[PROC. - Nitric Oxide Measurement 24](#_vavyb1h29p)

[PROC. - Oscillatory positive expiratory pressure 24](#_uotzzruwpbc6)

[PROC. - Sinus Surgery 24](#_og4bofb149l0)

[PROC. - Detection of Micro-organsms 26](#_ikk89ie0sqgy)

[PROC. - Sputum Induction 27](#_boctugcd2f2w)

[PROC. - Steroid Injection 27](#_uv8by76zvoxx)

[PROC. - Sweat Chloride Test 27](#_elv8yx1wyc4m)

[PROC. - Tonsil surgery 27](#_q1u6iboxvd11)

[PROC. - Tympanoplasty 28](#_bujm5t17kbiy)

[**DRUG CODES 28**](#_8a4e6irj8fuo)

[DRUG - Antibiotics 29](#_9j5mvp53jymo)

[DRUG - Antidepressant 30](#_ey5413331l9c)

[DRUG - Antifungals 30](#_75mhnmso2slw)

[DRUG - Antihistamine 30](#_bcglj4enn8s6)

[DRUG - Anti-inflammatory (Steroid) 30](#_5z81xbebz9ic)

[DRUG - Anti-inflammatory (Non-Steroid) 30](#_vs8tmt8031q1)

[DRUG - Bronchodilators 31](#_kfu50jglmv4v)

[DRUG - Decongestant 31](#_y98x4ucgvnrw)

[DRUG - Expectorant 31](#_n9qnxg10pid9)

[DRUG - Mucolytics 31](#_53jno6rz7o0v)

#

# **INTRODUCTION**

This document describes how we are coding and capturing features for computational analysis with machine learning. At the current time this data is expressed as a list of codes grouped into categories. We have defined 28 categories for Diagnoses, 28 for Procedures, and 12 for Drug Prescriptions. These are fully described below.

# **DIAGNOSIS CATEGORIES AND MEASURES**

We split the main categories into diagnoses pertaining to (A) issues of the airway and of breathing; (B) congenital malformations; (C) broad categories of diagnoses and possible misdiagnoses; (D) ear, nose, and sinus-related infections and issues; (E) fertility issues; (F) quality of life measures to characterize how the disease impacts patients experience more deeply.

Each category is assigned a feature type in parentheses and **COUNT**, **COVERAGE**, or **FIRST** in a way that determines how the feature is computed.

**COUNT** features are simply the number of separate days that a diagnosis in that category occurs in the course of a year. These groups of diagnosis codes are typically serious events (such as a collapsed lung, a pneumonia diagnosis, or the presence of COVID-19).

**COVERAGE** features are the number of separate two-week periods in which a diagnosis from that category appears over the course of a year. These events are thought to be more chronic and interconnected, so that we treat one occurrence over the two weeks the same as 5 similar events in the same time period.

**FIRST** features are binary features that are set to 0.0 before they first occur in the longitudinal data and 1.0 after that. This coding scheme denotes the presence of conditions that don’t change over time (e.g,. Situs Inversus or other congenital malformations, bronchiectasis, etc).

The categories of diagnostic features being used are:

- AIRWAY - Atelectasis (COUNT)
- AIRWAY - Breathing Issues (COVERAGE)
- AIRWAY - Bronchiectasis (FIRST)
- AIRWAY - Bronchitis (COVERAGE)
- AIRWAY - Cough (COVERAGE)
- AIRWAY - Pneumonia (COUNT)
- AIRWAY - Serious Pulmonology Events (COUNT)
- AIRWAY - Upper Airway Infections (COVERAGE)
- CM - Congenital Malformations of Heart (FIRST)
- CM - Congenital Malformations of Spleen (FIRST)
- CM - Congenital Malformations of Other Organs (FIRST)
- CM - Unspecified Congenital Malformations (FIRST)
- CM - Situs Inversus (FIRST)
- DIAG. - Asthma (COUNT)
- DIAG. - COVID-19 (COUNT)
- DIAG. - Cystic Fibrosis (COUNT)
- DIAG. - Pulmonary Diagnoses (COUNT)
- DIAG. - Q348 - Congenital Malf. Resp. Sys. (Specific) (FIRST)
- EAR - Hearing Loss (COUNT)
- EAR - Otitis media (COVERAGE)
- EAR - Otorrhea (COVERAGE)
- FERTILITY - Infertility (COVERAGE)
- IMMUNE SYSTEM - Immunodeficiency (COVERAGE)
- NOSE - Nasal Congestion (COVERAGE)
- NOSE - Nasal Polyps (COVERAGE)
- NOSE - Rhinitis (COVERAGE)
- QOL - Depression or Anxiety (COVERAGE)
- SINUS - Chronic Sinusitis (COVERAGE)

#

# **ICD DIAGNOSIS CODES**

## AIRWAY - Atelectasis

J9811 - Atelectasis

## AIRWAY - Breathing Issues

78606 - Tachypnea

R062 - Wheezing

R069 - Unspecified Abnormalities Of Breathing

R0600 - Dyspnea, Unspecified

R0602 - Shortness Of Breath

R0682 - Tachypnea, Not Elsewhere Classified

R0689 - Other Abnormalities Of Breathing

## AIRWAY - Bronchiectasis

4940 - Bronchiectasis Without Acute Exacerbation

4941 - Bronchiectasis With Acute Exacerbation

74861 - Congenital Bronchiectasis (1.8k)

J47 - Bronchiectasis (1.3k)

J470 - Bronchiectasis With Acute Lower Respiratory Infection

J471 - Bronchiectasis With (Acute) Exacerbation

J479 - Bronchiectasis, Uncomplicated

Q334 - Congenital Bronchiectasis (3.5k)

## AIRWAY - Bronchitis

466 - Acute bronchitis and bronchiolitis

490 - Bronchitis, not specified as acute or chronic

491 - Chronic bronchitis

4660 - Acute bronchitis

4910 - Simple chronic bronchitis

4910 - Simple Chronic Bronchitis

4911 - Mucopurulent Chronic Bronchitis

4912 - Obstructive chronic bronchitis

4918 - Other Chronic Bronchitis

4919 - Unspecified Chronic Bronchitis

5060 - Bronchitis and pneumonitis due to fumes and vapors

49120 - Obstructive Chronic Bronchitis Without Exacerbation

49121 - Obstructive Chronic Bronchitis With (Acute) Exacerbation

49122 - Obstructive Chronic Bronchitis With Acute Bronchitis

J20 - Acute bronchitis

J40 - Bronchitis, not specified as acute or chronic

J41 - Simple And Mucopurulent Chronic Bronchitis

J42 - Unspecified Chronic Bronchitis

J200 - Acute bronchitis due to Mycoplasma pneumoniae

J201 - Acute bronchitis due to Hemophilus influenzae

J202 - Acute bronchitis due to streptococcus

J203 - Acute bronchitis due to coxsackievirus

J204 - Acute bronchitis due to parainfluenza virus

J205 - Acute bronchitis due to respiratory syncytial virus

J206 - Acute bronchitis due to rhinovirus

J207 - Acute bronchitis due to echovirus

J208 - Acute bronchitis due to other specified organisms

J209 - Acute bronchitis, unspecified

J410 - Simple Chronic Bronchitis

J411 - Mucopurulent Chronic Bronchitis

J418 - Mixed Simple And Mucopurulent Chronic Bronchitis

J680 - Bronchitis and pneumonitis due to chemicals, gases, fumes and vapors

## AIRWAY - Cough

7862 - Cough

49382 - Cough Variant Asthma

G4483 - Primary Cough Headache

J45991 - Cough Variant Asthma

R05 - Cough

R052 - Subacute cough

R053 - Chronic cough

## AIRWAY - Serious Pulmonology Events

9601 - Acute Respiratory Failure With Hypoxia

51851 - Acute Respiratory Failure Following Trauma And Surgery

51853 - Acute And Chronic Respiratory Failure Following Trauma And Surgery

51881 - Acute Respiratory Failure

51883 - Chronic Respiratory Failure

51884 - Acute And Chronic Respiratory Failure

J80 - Acute Respiratory Distress Syndrome

J90 - Pleural Effusion, Not Elsewhere Classified

J96 - Respiratory Failure, Not Elsewhere Classified

J810 - Acute Pulmonary Edema

J960 - Acute Respiratory Failure (5.7k)

J961 - Chronic Respiratory Failure (3.1k)

J962 - Acute And Chronic Respiratory Failure (1.7k)

J969 - Respiratory Failure, Unspecified (1.6k)

J9582 - Postprocedural Respiratory Failure (2.1k)

J9600 - Acute Respiratory Failure, Unspecified Whether With Hypoxia Or Hypercapnia

J9601 - Acute Respiratory Failure With Hypoxia

J9602 - Acute Respiratory Failure With Hypercapnia

J9610 - Chronic Respiratory Failure, Unspecified Whether With Hypoxia Or Hypercapnia

J9611 - Chronic Respiratory Failure With Hypoxia

J9612 - Chronic Respiratory Failure With Hypercapnia

J9620 - Acute And Chronic Respiratory Failure, Unspecified Whether With Hypoxia Or Hypercapnia

J9621 - Acute And Chronic Respiratory Failure With Hypoxia

J9621 - Acute And Chronic Respiratory Failure With Hypoxia

J9622 - Acute And Chronic Respiratory Failure With Hypercapnia

J9690 - Respiratory Failure, Unspecified, Unspecified Whether With Hypoxia Or Hypercapnia

J9691 - Respiratory Failure, Unspecified With Hypoxia

J9692 - Respiratory Failure, Unspecified With Hypercapnia

J95821 - Acute Postprocedural Respiratory Failure

J95822 - Acute And Chronic Postprocedural Respiratory Failure

R0603 - Acute Respiratory Distress

## AIRWAY - Pneumonia

481 - Pneumococcal Pneumonia [Streptococcus Pneumoniae Pneumonia]

486 - Pneumonia, Organism Unspecified

4829 - Bacterial Pneumonia, Unspecified

4838 - Pneumonia Due To Other Specified Organism

J13 - Pneumonia Due To Streptococcus Pneumoniae

J150 - Pneumonia Due To Klebsiella Pneumoniae

J151 - Pneumonia Due To Pseudomonas

J156 - Pneumonia Due To Other Gram-Negative Bacteria

J157 - Pneumonia Due To Mycoplasma Pneumoniae

J158 - Pneumonia Due To Other Specified Bacteria

J159 - Unspecified Bacterial Pneumonia

J168 - Pneumonia Due To Other Specified Infectious Organisms

J181 - Lobar Pneumonia, Unspecified Organism

J188 - Other Pneumonia, Unspecified Organism

J189 - Pneumonia, Unspecified Organism

J15212 - Pneumonia Due To Methicillin Resistant Staphylococcus Aureus

V1261 - Personal History Of Pneumonia (Recurrent)

Z8701 - Personal History Of Pneumonia (Recurrent)

## AIRWAY - Upper Airway Infections

4658 - Acute Upper Respiratory Infections Of Other Multiple Sites

4659 - Acute Upper Respiratory Infections Of Unspecified Site

J069 - Acute Upper Respiratory Infection, Unspecified

## CM - Congenital Malformations of Heart

I517 - Cardiomegaly

Q20 - Congenital Malformations Of Cardiac Chambers And Connections (3.5k)

Q21 - Congenital Malformations Of Cardiac Septa (3.4k)

Q24 - Other Congenital Malformations Of Heart (4.7k)

Q204 - Double Inlet Ventricle

Q208 - Other Congenital Malformations Of Cardiac Chambers And Connections

Q209 - Congenital Malformation Of Cardiac Chambers And Connections, Unspecified

Q210 - Ventricular Septal Defect

Q211 - Atrial Septal Defect

Q212 - Atrioventricular Septal Defect

Q213 - Tetralogy Of Fallot

Q214 - Aortopulmonary Septal Defect

Q218 - Other Congenital Malformations Of Cardiac Septa

Q219 - Congenital Malformation Of Cardiac Septum, Unspecified

Q238 - Other Congenital Malformations Of Aortic And Mitral Valves

Q240 - Dextrocardia

Q241 - Levocardia

Q242 - Cor Triatriatum (5.6k)

Q243 - Pulmonary Infundibular Stenosis

Q244 - Congenital Subaortic Stenosis

Q245 - Malformation Of Coronary Vessels

Q246 - Congenital Heart Block

Q248 - Other Specified Congenital Malformations Of Heart

Q249 - Congenital Malformation Of Heart, Unspecified

## CM - Other Congenital Malformations

Q89 - Other Congenital Malformations, Not Elsewhere Classified (3.5k)

Q453 - Other Congenital Malformations Of Pancreas And Pancreatic Duct

Q638 - Other Specified Congenital Malformations Of Kidney

Q890 - Congenital Absence And Malformations Of Spleen (14)

Q891 - Congenital Malformations Of Adrenal Gland

Q892 - Congenital Malformations Of Other Endocrine Glands

Q897 - Multiple Congenital Malformations, Not Elsewhere Classified

Q898 - Other Specified Congenital Malformations

Q899 - Congenital Malformation, Unspecified

Q8901 - Asplenia (Congenital)

Q8909 - Congenital Malformations Of Spleen

## CM - Situs Inversus

Q893 - Situs Inversus

## DIAG. - Asthma

49300 - Extrinsic Asthma, Unspecified

49301 - Extrinsic Asthma With Status Asthmaticus

49302 - Extrinsic Asthma With (Acute) Exacerbation

49310 - Intrinsic Asthma, Unspecified

49320 - Chronic Obstructive Asthma, Unspecified

49382 - Cough Variant Asthma

49390 - Asthma, Unspecified Type, Unspecified

49391 - Asthma, Unspecified Type, With Status Asthmaticus

49392 - Asthma, Unspecified Type, With (Acute) Exacerbation

J455 - Severe Persistent Asthma (790)

J4520 - Mild Intermittent Asthma, Uncomplicated

J4521 - Mild Intermittent Asthma With (Acute) Exacerbation

J4522 - Mild Intermittent Asthma With Status Asthmaticus

J4530 - Mild Persistent Asthma, Uncomplicated

J4531 - Mild Persistent Asthma With (Acute) Exacerbation

J4540 - Moderate Persistent Asthma, Uncomplicated

J4541 - Moderate Persistent Asthma With (Acute) Exacerbation

J4542 - Moderate Persistent Asthma With Status Asthmaticus

J4550 - Severe Persistent Asthma, Uncomplicated

J4550 - Severe Persistent Asthma, Uncomplicated

J4551 - Severe Persistent Asthma With (Acute) Exacerbation

J4551 - Severe Persistent Asthma With (Acute) Exacerbation

J4552 - Severe Persistent Asthma With Status Asthmaticus

J45901 - Unspecified Asthma With (Acute) Exacerbation

J45902 - Unspecified Asthma With Status Asthmaticus

J45909 - Unspecified Asthma, Uncomplicated

J45998 - Other Asthma

Z825 - Family History Of Asthma And Other Chronic Lower Respiratory Diseases

## DIAG. - COVID-19

U071 - Covid-19

## DIAG. - Cystic Fibrosis

27702 - Cystic Fibrosis With Pulmonary Manifestations

E84 - Cystic Fibrosis (1.5k)

E840 - Cystic Fibrosis With Pulmonary Manifestations

E849 - Cystic Fibrosis, Unspecified

## DIAG. - Pulmonary Diagnoses

J984 - Other Disorders Of Lung

J988 - Other Specified Respiratory Disorders

J9809 - Other Diseases Of Bronchus, Not Elsewhere Classified

R918 - Other Nonspecific Abnormal Finding Of Lung Field

Z8709 - Personal History Of Other Diseases Of The Respiratory System

## DIAG. - Q348 - Congenital Malf. Resp. Sys. (Specific)

Q348 - Other Specified Congenital Malformations Of Respiratory System (8.6k)

## EAR - Otitis media

3813 - Other And Unspecified Chronic Nonsuppurative Otitis Media

3814 - Nonsuppurative Otitis Media, Not Specified As Acute Or Chronic

3821 - Chronic Tubotympanic Suppurative Otitis Media

3822 - Chronic Atticoantral Suppurative Otitis Media

3823 - Unspecified Chronic Suppurative Otitis Media

3829 - Unspecified Otitis Media

38100 - Acute Nonsuppurative Otitis Media, Unspecified

38101 - Acute Serous Otitis Media

38110 - Chronic Serous Otitis Media, Simple Or Unspecified

38119 - Other Chronic Serous Otitis Media

38120 - Chronic Mucoid Otitis Media, Simple Or Unspecified

38129 - Other Chronic Mucoid Otitis Media

38200 - Acute Suppurative Otitis Media Without Spontaneous Rupture Of Eardrum

H663X1 - Other Chronic Suppurative Otitis Media, Right Ear

H663X2 - Other Chronic Suppurative Otitis Media, Left Ear

H663X3 - Other Chronic Suppurative Otitis Media, Bilateral

H663X9 - Other Chronic Suppurative Otitis Media, Unspecified Ear

H6500 - Acute Serous Otitis Media, Unspecified Ear

H6501 - Acute Serous Otitis Media, Right Ear

H6502 - Acute Serous Otitis Media, Left Ear

H6503 - Acute Serous Otitis Media, Bilateral

H6520 - Chronic Serous Otitis Media, Unspecified Ear

H6521 - Chronic Serous Otitis Media, Right Ear

H6522 - Chronic Serous Otitis Media, Left Ear

H6523 - Chronic Serous Otitis Media, Bilateral

H6530 - Chronic Mucoid Otitis Media, Unspecified Ear

H6531 - Chronic Mucoid Otitis Media, Right Ear

H6532 - Chronic Mucoid Otitis Media, Left Ear

H6533 - Chronic Mucoid Otitis Media, Bilateral

H6590 - Unspecified Nonsuppurative Otitis Media, Unspecified Ear

H6591 - Unspecified Nonsuppurative Otitis Media, Right Ear

H6592 - Unspecified Nonsuppurative Otitis Media, Left Ear

H6593 - Unspecified Nonsuppurative Otitis Media, Bilateral

H6611 - Chronic Tubotympanic Suppurative Otitis Media, Right Ear

H6612 - Chronic Tubotympanic Suppurative Otitis Media, Left Ear

H6623 - Chronic Atticoantral Suppurative Otitis Media, Bilateral

H6641 - Suppurative Otitis Media, Unspecified, Right Ear

H6642 - Suppurative Otitis Media, Unspecified, Left Ear

H6643 - Suppurative Otitis Media, Unspecified, Bilateral

H6690 - Otitis Media, Unspecified, Unspecified Ear

H6691 - Otitis Media, Unspecified, Right Ear

H6692 - Otitis Media, Unspecified, Left Ear

H6693 - Otitis Media, Unspecified, Bilateral

H65191 - Other Acute Nonsuppurative Otitis Media, Right Ear

H65192 - Other Acute Nonsuppurative Otitis Media, Left Ear

H65193 - Other Acute Nonsuppurative Otitis Media, Bilateral

H65413 - Chronic Allergic Otitis Media, Bilateral

H65491 - Other Chronic Nonsuppurative Otitis Media, Right Ear

H65492 - Other Chronic Nonsuppurative Otitis Media, Left Ear

H65493 - Other Chronic Nonsuppurative Otitis Media, Bilateral

H65499 - Other Chronic Nonsuppurative Otitis Media, Unspecified Ear

H66001 - Acute Suppurative Otitis Media Without Spontaneous Rupture Of Ear Drum, Right Ear

H66002 - Acute Suppurative Otitis Media Without Spontaneous Rupture Of Ear Drum, Left Ear

H66003 - Acute Suppurative Otitis Media Without Spontaneous Rupture Of Ear Drum, Bilateral

H66006 - Acute Suppurative Otitis Media Without Spontaneous Rupture Of Ear Drum, Recurrent, Bilateral

## EAR - Hearing Loss

389 - Hearing loss

3882 - Sudden hearing loss, unspecified

3890 - Conductive hearing loss

3891 - Sensorineural hearing loss

3892 - Mixed conductive and sensorineural hearing loss

3898 - Other specified forms of hearing loss

3899 - Unspecified hearing loss

31534 - Speech and language developmental delay due to hearing loss

38812 - Noise-induced hearing loss

38900 - Conductive hearing loss, unspecified

38901 - Conductive hearing loss, external ear

38902 - Conductive hearing loss, tympanic membrane

38903 - Conductive hearing loss, middle ear

38904 - Conductive hearing loss, inner ear

38905 - Conductive hearing loss, unilateral

38906 - Conductive hearing loss, bilateral

38908 - Conductive hearing loss of combined types

38910 - Sensorineural hearing loss, unspecified

38911 - Sensory hearing loss, bilateral

38912 - Neural hearing loss, bilateral

38913 - Neural hearing loss, unilateral

38914 - Central hearing loss

38915 - Sensorineural hearing loss, unilateral

38916 - Sensorineural hearing loss, asymmetrical

38917 - Sensory hearing loss, unilateral

38918 - Sensorineural hearing loss, bilateral

38920 - Mixed hearing loss, unspecified

38921 - Mixed hearing loss, unilateral

38922 - Mixed hearing loss, bilateral

F804 - Speech and language development delay due to hearing loss

H90 - Conductive and sensorineural hearing loss

H90A - Conductive and sensorineural hearing loss with restricted hearing on the contralateral side

H90A1 - Conductive hearing loss, unilateral, with restricted hearing on the contralateral side

H90A2 - Sensorineural hearing loss, unilateral, with restricted hearing on the contralateral side

H90A3 - Mixed conductive and sensorineural hearing loss, unilateral with restricted hearing on the contralateral side

H90A11 - Conductive hearing loss, unilateral, right ear with restricted hearing on the contralateral side

H90A12 - Conductive hearing loss, unilateral, left ear with restricted hearing on the contralateral side

H90A21 - Sensorineural hearing loss, unilateral, right ear, with restricted hearing on the contralateral side

H90A22 - Sensorineural hearing loss, unilateral, left ear, with restricted hearing on the contralateral side

H90A31 - Mixed conductive and sensorineural hearing loss, unilateral, right ear with restricted hearing on the contralateral side

H90A32 - Mixed conductive and sensorineural hearing loss, unilateral, left ear with restricted hearing on the contralateral side

H91 - Other and unspecified hearing loss

H900 - Conductive hearing loss, bilateral

H901 - Conductive hearing loss, unilateral with unrestricted hearing on the contralateral side

H902 - Conductive hearing loss, unspecified

H903 - Sensorineural hearing loss, bilateral

H904 - Sensorineural hearing loss, unilateral with unrestricted hearing on the contralateral side

H905 - Unspecified sensorineural hearing loss

H906 - Mixed conductive and sensorineural hearing loss, bilateral

H907 - Mixed conductive and sensorineural hearing loss, unilateral with unrestricted hearing on the contralateral side

H908 - Mixed conductive and sensorineural hearing loss, unspecified

H910 - Ototoxic hearing loss

H912 - Sudden idiopathic hearing loss

H918 - Other specified hearing loss

H918X - Other specified hearing loss

H918X1 - Other specified hearing loss, right ear

H918X2 - Other specified hearing loss, left ear

H918X3 - Other specified hearing loss, bilateral

H918X9 - Other specified hearing loss, unspecified ear

H919 - Unspecified hearing loss

H9011 - Conductive hearing loss, unilateral, right ear, with unrestricted hearing on the contralateral side

H9012 - Conductive hearing loss, unilateral, left ear, with unrestricted hearing on the contralateral side

H9041 - Sensorineural hearing loss, unilateral, right ear, with unrestricted hearing on the contralateral side

H9042 - Sensorineural hearing loss, unilateral, left ear, with unrestricted hearing on the contralateral side

H9071 - Mixed conductive and sensorineural hearing loss, unilateral, right ear, with unrestricted hearing on the contralateral side

H9072 - Mixed conductive and sensorineural hearing loss, unilateral, left ear, with unrestricted hearing on the contralateral side

H9101 - Ototoxic hearing loss, right ear

H9102 - Ototoxic hearing loss, left ear

H9103 - Ototoxic hearing loss, bilateral

H9109 - Ototoxic hearing loss, unspecified ear

H9120 - Sudden idiopathic hearing loss, unspecified ear

H9121 - Sudden idiopathic hearing loss, right ear

H9122 - Sudden idiopathic hearing loss, left ear

H9123 - Sudden idiopathic hearing loss, bilateral

H9190 - Unspecified hearing loss, unspecified ear

H9191 - Unspecified hearing loss, right ear

H9192 - Unspecified hearing loss, left ear

H9193 - Unspecified hearing loss, bilateral

P096 - Abnormal findings on neonatal screening for neonatal hearing loss

V192 - Family history of deafness or hearing loss

Z822 - Family history of deafness and hearing loss

## EAR - Otorrhea

38860 - Otorrhea, Unspecified

38861 - Cerebrospinal Fluid Otorrhea (5.9k)

38869 - Other Otorrhea

H921 - Otorrhea (460)

H9210 - Otorrhea, Unspecified Ear

H9211 - Otorrhea, Right Ear

H9212 - Otorrhea, Left Ear

H9213 - Otorrhea, Bilateral

## FERTILITY - Infertility

6069 - Male Infertility, Unspecified

6282 - Infertility, Female, Of Tubal Origin

6288 - Infertility, Female, Of Other Specified Origin

6289 - Infertility, Female, Of Unspecified Origin

N46 - Male Infertility (3.8k)

N97 - Female Infertility (4.2k)

N468 - Other Male Infertility

N469 - Male Infertility, Unspecified

N971 - Female Infertility Of Tubal Origin

N978 - Female Infertility Of Other Origin

N979 - Female Infertility, Unspecified

## IMMUNE SYSTEM - Immunodeficiency

042 - Human Immunodeficiency Virus [Hiv] Disease

07953 - Human Immunodeficiency Virus, Type 2 [Hiv-2]

27901 - Selective Iga Immunodeficiency

27906 - Common Variable Immunodeficiency

27910 - Immunodeficiency With Predominant T-Cell Defect, Unspecified

79571 - Nonspecific Serologic Evidence Of Human Immunodeficiency Virus [Hiv]

B20 - Human Immunodeficiency Virus [Hiv] Disease

B9735 - Human Immunodeficiency Virus, Type 2 [Hiv 2] As The Cause Of Diseases Classified Elsewhere

D809 - Immunodeficiency With Predominantly Antibody Defects, Unspecified

D819 - Combined Immunodeficiency, Unspecified

D823 - Immunodeficiency Following Hereditary Defective Response To Epstein-Barr Virus

D830 - Common Variable Immunodeficiency With Predominant Abnormalities Of B-Cell Numbers And Function

D831 - Common Variable Immunodeficiency With Predominant Immunoregulatory T-Cell Disorders

D839 - Common Variable Immunodeficiency, Unspecified

D849 - Immunodeficiency, Unspecified

D8481 - Immunodeficiency Due To Conditions Classified Elsewhere

D84821 - Immunodeficiency Due To Drugs

D84822 - Immunodeficiency Due To External Causes

O9872 - Human Immunodeficiency Virus [Hiv] Disease Complicating Childbirth

O98712 - Human Immunodeficiency Virus [Hiv] Disease Complicating Pregnancy, Second Trimester

O98713 - Human Immunodeficiency Virus [Hiv] Disease Complicating Pregnancy, Third Trimester

O98719 - Human Immunodeficiency Virus [Hiv] Disease Complicating Pregnancy, Unspecified Trimester (9.6k)

R75 - Inconclusive Laboratory Evidence Of Human Immunodeficiency Virus [Hiv]

V08 - Asymptomatic Human Immunodeficiency Virus [Hiv] Infection Status

V6544 - Human Immunodeficiency Virus (Hiv) Counseling

Z21 - Asymptomatic Human Immunodeficiency Virus [Hiv] Infection Status

Z114 - Encounter For Screening For Human Immunodeficiency Virus [Hiv]

Z206 - Contact With And (Suspected) Exposure To Human Immunodeficiency Virus [Hiv]

Z717 - Human Immunodeficiency Virus [Hiv] Counseling

Z830 - Family History Of Human Immunodeficiency Virus [Hiv] Disease

## NOSE - Nasal Congestion

R0981 - Nasal Congestion

## NOSE - Nasal Polyps

4719 - Unspecified Nasal Polyp

4710 - Polyp Of Nasal Cavity

J33 - Nasal Polyp (2.7k)

J330 - Polyp Of Nasal Cavity

J339 - Nasal Polyp, Unspecified

## NOSE - Rhinitis

4720 - Chronic Rhinitis

J300 - Vasomotor Rhinitis

J31 - Chronic Rhinitis, Nasopharyngitis And Pharyngitis (3.9k)

J310 - Chronic Rhinitis

## QOL - Depression or Anxiety

311 - Depressive Disorder, Not Elsewhere Classified

29620 - Major Depressive Affective Disorder, Single Episode, Unspecified

29630 - Major Depressive Affective Disorder, Recurrent Episode, Unspecified

29632 - Major Depressive Affective Disorder, Recurrent Episode, Moderate

29633 - Major Depressive Affective Disorder, Recurrent Episode, Severe, Without Mention Of Psychotic

30000 - Anxiety State, Unspecified

30002 - Generalized Anxiety Disorder

30009 - Other Anxiety States

30924 - Adjustment Disorder With Anxiety

30928 - Adjustment Disorder With Mixed Anxiety And Depressed Mood

30928 - Adjustment Disorder With Mixed Anxiety And Depressed Mood

F32A - Depression, unspecified

F064 - Anxiety Disorder Due To Known Physiological Condition

F320 - Major Depressive Disorder, Single Episode, Mild

F320 - Major Depressive Disorder, Single Episode, Mild

F321 - Major Depressive Disorder, Single Episode, Moderate

F322 - Major Depressive Disorder, Single Episode, Severe Without Psychotic Features

F325 - Major Depressive Disorder, Single Episode, In Full Remission

F329 - Major Depressive Disorder, Single Episode, Unspecified

F330 - Major Depressive Disorder, Recurrent, Mild

F331 - Major Depressive Disorder, Recurrent, Moderate

F332 - Major Depressive Disorder, Recurrent Severe Without Psychotic Features

F339 - Major Depressive Disorder, Recurrent, Unspecified

F410 - Panic Disorder [Episodic Paroxysmal Anxiety]

F411 - Generalized Anxiety Disorder

F413 - Other Mixed Anxiety Disorders

F418 - Other Specified Anxiety Disorders

F419 - Anxiety Disorder, Unspecified

F3289 - Other Specified Depressive Episodes

F3341 - Major Depressive Disorder, Recurrent, In Partial Remission

F4321 - Adjustment Disorder With Depressed Mood

F4321 - Adjustment Disorder With Depressed Mood

F4322 - Adjustment Disorder With Anxiety

F4323 - Adjustment Disorder With Mixed Anxiety And Depressed Mood

F4323 - Adjustment Disorder With Mixed Anxiety And Depressed Mood

V790 - [Icd10] Driver Of Bus Injured In Collision ... (Or) [Icd9] Screening For Depression...

## SINUS - Chronic Sinusitis

4730 - Chronic Maxillary Sinusitis

4731 - Chronic Frontal Sinusitis

4732 - Chronic Ethmoidal Sinusitis

4733 - Chronic Sphenoidal Sinusitis

4738 - Other Chronic Sinusitis

4739 - Unspecified Sinusitis (Chronic)

J32 - Chronic Sinusitis (3.6k)

J320 - Chronic Maxillary Sinusitis

J321 - Chronic Frontal Sinusitis

J322 - Chronic Ethmoidal Sinusitis

J323 - Chronic Sphenoidal Sinusitis

J328 - Other Chronic Sinusitis

J329 - Chronic Sinusitis, Unspecified

# **PROCEDURE CATEGORIES**

We did not denote procedure categories into groups. Procedure codes are assigned feature types in the same way

- PROC. - Acapella/Flutter: (COUNT)
- PROC. - Adenoidectomy: (COUNT)
- PROC. - BAHA: (COUNT)
- PROC. - Bronchoalveoloar lavage: (COVERAGE)
- PROC. - Chest Wall Manipulation: (COVERAGE)
- PROC. - Chest X-Rays: (COVERAGE)
- PROC. - Cochlear Implant Maintenance: (COVERAGE)
- PROC. - Cochlear Implant Placement: (COUNT)
- PROC. - Detection of Micro-organsms: (COVERAGE)
- PROC. - Diagnosic EM: (FIRST)
- PROC. - Endoscopic airway exam: (COVERAGE)
- PROC. - Genetic Testing: (COUNT)
- PROC. - Hearing aid Fitting + Maintenance: (COVERAGE)
- PROC. - Hospitalization: (COUNT)
- PROC. - IPV: (COUNT)
- PROC. - Lung Function Measurement: (COVERAGE)
- PROC. - Lung Transplant: (COUNT)
- PROC. - Mastoidectomy: (COUNT)
- PROC. - Myringotomy Tubes: (COUNT)
- PROC. - Nasal Biopsy: (COUNT)
- PROC. - Nitric Oxide Measurement: (COVERAGE)
- PROC. - Oscillatory positive expiratory pressure: (COUNT)
- PROC. - Sinus Surgery: (COVERAGE)
- PROC. - Sputum Induction: (COVERAGE)
- PROC. - Steroid Injection: (COVERAGE)
- PROC. - Sweat Chloride Test: (COUNT)
- PROC. - Tonsil surgery: (COUNT)
- PROC. - Tympanoplasty: (COUNT)

# **PROCEDURE CODES**

## PROC. - Acapella/Flutter

S8185 - Flutter device

## PROC. - Adenoidectomy

283 - Tonsillectomy with adenoidectomy

286 - Adenoidectomy without tonsillectomy

287 - Control of hemorrhage after tonsillectomy and adenoidectomy

42830 - Removal of adenoids patient younger than age 12, initial procedure

42831 - Removal of adenoids patient age 12 or over, initial procedure

42835 - Removal of adenoids patient younger than age 12, secondary procedure

42836 - Removal of adenoids patient age 12 or over, secondary procedure

## PROC. - BAHA

69710 - Implantation or replacement of temporal bone conduction hearing device

69711 - Removal or repair of temporal bone conduction hearing device

## PROC. - Bronchoalveoloar lavage

0B9C00Z - Drainage of Right Upper Lung Lobe with Drainage Device, Open Approach

0B9C0ZX - Drainage of Right Upper Lung Lobe, Open Approach, Diagnostic

0B9C3ZX - Drainage of Right Upper Lung Lobe, Percutaneous Approach, Diagnostic

0B9C3ZZ - Drainage of Right Upper Lung Lobe, Percutaneous Approach

0B9C4ZX - Drainage of Right Upper Lung Lobe, Percutaneous Endoscopic Approach, Diagnostic

0B9C4ZZ - Drainage of Right Upper Lung Lobe, Percutaneous Endoscopic Approach

0B9C7ZX - Drainage of Right Upper Lung Lobe, Via Natural or Artificial Opening, Diagnostic

0B9C7ZZ - Drainage of Right Upper Lung Lobe, Via Natural or Artificial Opening

0B9C8ZX - Drainage of Right Upper Lung Lobe, Via Natural or Artificial Opening Endoscopic, Diagnostic

0B9C8ZZ - Drainage of Right Upper Lung Lobe, Via Natural or Artificial Opening Endoscopic

0B9C30Z - Drainage of Right Upper Lung Lobe with Drainage Device, Percutaneous Approach

0B9C40Z - Drainage of Right Upper Lung Lobe with Drainage Device, Percutaneous Endoscopic Approach

0B9C70Z - Drainage of Right Upper Lung Lobe with Drainage Device, Via Natural or Artificial Opening

0B9C80Z - Drainage of Right Upper Lung Lobe with Drainage Device, Via Natural or Artificial Opening Endoscopic

0B9D00Z - Drainage of Right Middle Lung Lobe with Drainage Device, Open Approach

0B9D0ZX - Drainage of Right Middle Lung Lobe, Open Approach, Diagnostic

0B9D0ZZ - Drainage of Right Middle Lung Lobe, Open Approach

0B9D3ZX - Drainage of Right Middle Lung Lobe, Percutaneous Approach, Diagnostic

0B9D3ZZ - Drainage of Right Middle Lung Lobe, Percutaneous Approach

0B9D4ZX - Drainage of Right Middle Lung Lobe, Percutaneous Endoscopic Approach, Diagnostic

0B9D7ZX - Drainage of Right Middle Lung Lobe, Via Natural or Artificial Opening, Diagnostic

0B9D7ZZ - Drainage of Right Middle Lung Lobe, Via Natural or Artificial Opening

0B9D8ZX - Drainage of Right Middle Lung Lobe, Via Natural or Artificial Opening Endoscopic, Diagnostic

0B9D8ZZ - Drainage of Right Middle Lung Lobe, Via Natural or Artificial Opening Endoscopic

0B9D30Z - Drainage of Right Middle Lung Lobe with Drainage Device, Percutaneous Approach

0B9D40Z - Drainage of Right Middle Lung Lobe with Drainage Device, Percutaneous Endoscopic Approach

0B9D70Z - Drainage of Right Middle Lung Lobe with Drainage Device, Via Natural or Artificial Opening

0B9D80Z - Drainage of Right Middle Lung Lobe with Drainage Device, Via Natural or Artificial Opening Endoscopic

0B9F00Z - Drainage of Right Lower Lung Lobe with Drainage Device, Open Approach

0B9F0ZX - Drainage of Right Lower Lung Lobe, Open Approach, Diagnostic

0B9F0ZZ - Drainage of Right Lower Lung Lobe, Open Approach

0B9F3ZX - Drainage of Right Lower Lung Lobe, Percutaneous Approach, Diagnostic

0B9F3ZZ - Drainage of Right Lower Lung Lobe, Percutaneous Approach

0B9F4ZX - Drainage of Right Lower Lung Lobe, Percutaneous Endoscopic Approach, Diagnostic

0B9F4ZZ - Drainage of Right Lower Lung Lobe, Percutaneous Endoscopic Approach

0B9F7ZX - Drainage of Right Lower Lung Lobe, Via Natural or Artificial Opening, Diagnostic

0B9F7ZZ - Drainage of Right Lower Lung Lobe, Via Natural or Artificial Opening

0B9F8ZX - Drainage of Right Lower Lung Lobe, Via Natural or Artificial Opening Endoscopic, Diagnostic

0B9F8ZZ - Drainage of Right Lower Lung Lobe, Via Natural or Artificial Opening Endoscopic

0B9F30Z - Drainage of Right Lower Lung Lobe with Drainage Device, Percutaneous Approach

0B9F40Z - Drainage of Right Lower Lung Lobe with Drainage Device, Percutaneous Endoscopic Approach

0B9F70Z - Drainage of Right Lower Lung Lobe with Drainage Device, Via Natural or Artificial Opening

0B9F80Z - Drainage of Right Lower Lung Lobe with Drainage Device, Via Natural or Artificial Opening Endoscopic

0B9G00Z - Drainage of Left Upper Lung Lobe with Drainage Device, Open Approach

0B9G0ZX - Drainage of Left Upper Lung Lobe, Open Approach, Diagnostic

0B9G0ZZ - Drainage of Left Upper Lung Lobe, Open Approach

0B9G3ZX - Drainage of Left Upper Lung Lobe, Percutaneous Approach, Diagnostic

0B9G3ZZ - Drainage of Left Upper Lung Lobe, Percutaneous Approach

0B9G4ZX - Drainage of Left Upper Lung Lobe, Percutaneous Endoscopic Approach, Diagnostic

0B9G4ZZ - Drainage of Left Upper Lung Lobe, Percutaneous Endoscopic Approach

0B9G7ZX - Drainage of Left Upper Lung Lobe, Via Natural or Artificial Opening, Diagnostic

0B9G7ZZ - Drainage of Left Upper Lung Lobe, Via Natural or Artificial Opening

0B9G8ZX - Drainage of Left Upper Lung Lobe, Via Natural or Artificial Opening Endoscopic, Diagnostic

0B9G8ZZ - Drainage of Left Upper Lung Lobe, Via Natural or Artificial Opening Endoscopic

0B9G30Z - Drainage of Left Upper Lung Lobe with Drainage Device, Percutaneous Approach

0B9G40Z - Drainage of Left Upper Lung Lobe with Drainage Device, Percutaneous Endoscopic Approach

0B9G70Z - Drainage of Left Upper Lung Lobe with Drainage Device, Via Natural or Artificial Opening

0B9G80Z - Drainage of Left Upper Lung Lobe with Drainage Device, Via Natural or Artificial Opening Endoscopic

0B9J00Z - Drainage of Left Lower Lung Lobe with Drainage Device, Open Approach

0B9J0ZX - Drainage of Left Lower Lung Lobe, Open Approach, Diagnostic

0B9J0ZZ - Drainage of Left Lower Lung Lobe, Open Approach

0B9J3ZX - Drainage of Left Lower Lung Lobe, Percutaneous Approach, Diagnostic

0B9J3ZZ - Drainage of Left Lower Lung Lobe, Percutaneous Approach

0B9J4ZX - Drainage of Left Lower Lung Lobe, Percutaneous Endoscopic Approach, Diagnostic

0B9J4ZZ - Drainage of Left Lower Lung Lobe, Percutaneous Endoscopic Approach

0B9J7ZX - Drainage of Left Lower Lung Lobe, Via Natural or Artificial Opening, Diagnostic

0B9J7ZZ - Drainage of Left Lower Lung Lobe, Via Natural or Artificial Opening

0B9J8ZX - Drainage of Left Lower Lung Lobe, Via Natural or Artificial Opening Endoscopic, Diagnostic

0B9J8ZZ - Drainage of Left Lower Lung Lobe, Via Natural or Artificial Opening Endoscopic

0B9J30Z - Drainage of Left Lower Lung Lobe with Drainage Device, Percutaneous Approach

0B9J40Z - Drainage of Left Lower Lung Lobe with Drainage Device, Percutaneous Endoscopic Approach

0B9J70Z - Drainage of Left Lower Lung Lobe with Drainage Device, Via Natural or Artificial Opening

0B9J80Z - Drainage of Left Lower Lung Lobe with Drainage Device, Via Natural or Artificial Opening Endoscopic

31624 - Irrigation and suction of lung airways to obtain cells using an endoscope

88106 - Cell examination of body fluid, simple filter method

88108 - Cell examination of specimen, concentration technique

88112 - Cell examination of specimen, selective cellular enhancement technique

## PROC. - Cochlear Implant Placement

09HD05Z - Insertion of Single Channel Cochlear Prosthesis into Right Inner Ear, Open Approach

09HD06Z - Insertion of Multiple Channel Cochlear Prosthesis into Right Inner Ear, Open Approach

09HD35Z - Insertion of Single Channel Cochlear Prosthesis into Right Inner Ear, Percutaneous Approach

09HD36Z - Insertion of Multiple Channel Cochlear Prosthesis into Right Inner Ear, Percutaneous Approach

09HD45Z - Insertion of Single Channel Cochlear Prosthesis into Right Inner Ear, Percutaneous Endoscopic Approach

09HD46Z - Insertion of Multiple Channel Cochlear Prosthesis into Right Inner Ear, Percutaneous Endoscopic Approach

09HE05Z - Insertion of Single Channel Cochlear Prosthesis into Left Inner Ear, Open Approach

09HE06Z - Insertion of Multiple Channel Cochlear Prosthesis into Left Inner Ear, Open Approach

09HE35Z - Insertion of Single Channel Cochlear Prosthesis into Left Inner Ear, Percutaneous Approach

09HE36Z - Insertion of Multiple Channel Cochlear Prosthesis into Left Inner Ear, Percutaneous Approach

09HE45Z - Insertion of Single Channel Cochlear Prosthesis into Left Inner Ear, Percutaneous Endoscopic Approach

09HE46Z - Insertion of Multiple Channel Cochlear Prosthesis into Left Inner Ear, Percutaneous Endoscopic Approach

2096 - Implantation or replacement of cochlear prosthetic device, not otherwise specified

2097 - Implantation or replacement of cochlear prosthetic device, single channel

2098 - Implantation or replacement of cochlear prosthetic device, multiple channel

69714 - Temporal bone implantation of cochlear stimulating system, accessed through the skin

69715 - Removal of mastoid bone with implantation of cochlear stimulating system, accessed through the skin

69717 - Temporal bone replacement of cochlear stimulating system, accessed through the skin

69718 - Removal of mastoid bone with removal and replacement (accessed through the skin) of cochlear stimulating system

69930 - Implantation of cochlear device

92584 - Testing of nerve from ear to brain (cochlear)

92588 - Placement of ear probe for computerized cochlear assessment of repeated sounds with interpretation and report

## PROC. - Cochlear Implant Maintenance

92601 - Analysis and programming of inner ear (cochlear) implant, patient younger than 7 years of age

92602 - Analysis and reprogramming of inner ear (cochlear) implant, patient younger than 7 years of age

92603 - Analysis and programming of inner ear (cochlear) implant, patient age 7 years or older

92604 - Analysis and reprogramming of inner ear (cochlear) implant, patient age 7 years or older

F0B - Physical Rehabilitation and Diagnostic Audiology, Rehabilitation, Cochlear Implant Treatment

F0BZ0KZ - Cochlear Implant Rehabilitation Treatment using Audiovisual Equipment

F0BZ0PZ - Cochlear Implant Rehabilitation Treatment using Computer

F0BZ0YZ - Cochlear Implant Rehabilitation Treatment using Other Equipment

F0BZ01Z - Cochlear Implant Rehabilitation Treatment using Audiometer

F0BZ02Z - Cochlear Implant Rehabilitation Treatment using Sound Field / Booth

F0BZ09Z - Cochlear Implant Rehabilitation Treatment using Cochlear Implant Equipment

F00Z19Z - Speech Threshold Assessment using Cochlear Implant Equipment

F00Z29Z - Speech/Word Recognition Assessment using Cochlear Implant Equipment

F00Z59Z - Synthetic Sentence Identification Assessment using Cochlear Implant Equipment

F13Z09Z - Hearing Screening Assessment using Cochlear Implant Equipment

F13ZP9Z - Aural Rehabilitation Status Assessment using Cochlear Implant Equipment

F14Z0KZ - Cochlear Implant Assessment using Audiovisual Equipment

F14Z0LZ - Cochlear Implant Assessment using Assistive Listening Equipment

F14Z0PZ - Cochlear Implant Assessment using Computer

F14Z0YZ - Cochlear Implant Assessment using Other Equipment

F14Z0ZZ - Cochlear Implant Assessment

F14Z01Z - Cochlear Implant Assessment using Audiometer

F14Z02Z - Cochlear Implant Assessment using Sound Field / Booth

F14Z03Z - Cochlear Implant Assessment using Tympanometer

F14Z04Z - Cochlear Implant Assessment using Electroacoustic Immitance / Acoustic Reflex Equipment

F14Z05Z - Cochlear Implant Assessment using Hearing Aid Selection / Fitting / Test Equipment

F14Z07Z - Cochlear Implant Assessment using Electrophysiologic Equipment

F14Z09Z - Cochlear Implant Assessment using Cochlear Implant Equipment

L8614 - Cochlear device, includes all internal and external components

L8615 - Headset/headpiece for use with cochlear implant device, replacement

L8616 - Microphone for use with cochlear implant device, replacement

L8617 - Transmitting coil for use with cochlear implant device, replacement

L8618 - Transmitter cable for use with cochlear implant device or auditory osseointegrated device, replacement

L8619 - Cochlear implant, external speech processor and controller, integrated system, replacement

L8621 - Zinc air battery for use with cochlear implant device and auditory osseointegrated sound processors, replacement, each

L8622 - Alkaline battery for use with cochlear implant device, any size, replacement, each

L8623 - Lithium ion battery for use with cochlear implant device speech processor, other than ear level, replacement, each

L8624 - Lithium ion battery for use with cochlear implant or auditory osseointegrated device speech processor, ear level, replacement, each

L8625 - External recharging system for battery for use with cochlear implant or auditory osseointegrated device, replacement only, each

L8627 - Cochlear implant, external speech processor, component, replacement

L8628 - Cochlear implant, external controller component, replacement

L8629 - Transmitting coil and cable, integrated, for use with cochlear implant device, replacement

V5273 - Assistive listening device, for use with cochlear implant

## PROC. - Chest Wall Manipulation

94667 - Demonstration and/or evaluation of manual maneuvers to chest wall to assist movement of lung secretions

94668 - Manual maneuvers to chest wall to assist movement of lung secretions

94669 - Mechanical chest wall manipulation for improvement in lung function

A7025 - High Frequency Chest Wall Oscillation System Vest, Replacement For Use With Patient Owned Eq (430)

A7026 - High Frequency Chest Wall Oscillation System Hose, Replacement For Use With Patient Owned Eq (570)

E0483 - High frequency chest wall oscillation system, includes all accessories and supplies, each

## PROC. - Chest X-Rays

71010 - X-Ray Of Chest, 1 View, Front

71020 - X-Ray Of Chest, 2 Views, Front And Side

71020 - X-Ray Of Chest, 2 Views, Front And Side

71021 - X-Ray Of Chest, 2 Views, Front And Side

71022 - X-Ray Of Chest, 2 Views, Front And Side

71030 - X-Ray Of Chest, Minimum Of 4 Views

71035 - X-Ray Of Chest, Special Views

71045 - X-Ray Of Chest, 1 View

71046 - X-Ray Of Chest, 2 Views

71047 - X-Ray Of Chest, 3 Views

71048 - X-Ray Of Chest, Minimum Of 4 Views

71101 - X-Ray Of Ribs On One Side Of Body Including The Chest, Minimum Of 3 Views

71111 - X-Ray Of Both Sides Of The Ribs Including The Chest, Minimum Of 4 Views

71250 - Diagnostic CT scan of chest

71260 - Diagnostic CT scan of chest with contrast

74022 - Complete X-Ray Study Of Abdomen With Single X-Ray Of Chest

## PROC. - Diagnostic EM

88348 - Electron microscopy for diagnosis

## PROC. - Endoscopic airway exam

3321 - Bronchoscopy through artificial stoma

3322 - Fiber-optic bronchoscopy

3323 - Other bronchoscopy

31622 - Diagnostic examination of lung airways using an endoscope

31623 - Examination of lung airways using an endoscope

31625 - Biopsy of lung airways using an endoscope

C9751 - Bronchoscopy, rigid or flexible, transbronchial ablation of lesion(s) by microwave energy, including fluoroscopic guidance, when performed, with computed tomography acquisition(s) and 3-d rendering, computer-assisted, image-guided navigation, and endobronchial ultrasound (ebus) guided transtracheal and/or transbronchial sampling (eg, aspiration[s]/biopsy[ies]) and all mediastinal and/or hilar lymph node stations or structures and therapeutic intervention(s)

## PROC. - Genetic Testing

82016 - Chemical analysis for genetic disorder

82017 - Chemical test for genetic disorder

86352 - Analysis of cell function and analysis for genetic marker

88245 - Chromosome analysis for genetic defects, baseline Sister Chromatid Exchange (SCE), 20-25 cells

88261 - Chromosome analysis for genetic defects, count 5 cells

88262 - Chromosome analysis for genetic defects, count 15-20 cells

88264 - Chromosome analysis for genetic defects, analyze 20-25 cells

88271 - DNA testing for genetic defects

88273 - Chromosome analysis for genetic defects, analyze 10-30 cells

88274 - Chromosome analysis for genetic defects, analyze 25-99 cells

88280 - Chromosome analysis for genetic defects, additional karyotypes, each study

88285 - Chromosome analysis for genetic defects, additional cells counted, each study

88289 - Chromosome analysis for genetic defects, additional high resolution study

88291 - Interpretation and report of genetic testing

88365 - Genetic sequencing localization, initial procedure

## PROC. - Hearing aid Fitting + Maintenance

2095 - Implantation of electromagnetic hearing device

9548 - Fitting of hearing aid

92590 - Hearing aid examination and selection of one ear

92591 - Hearing aid examination and selection of both ears

92592 - Check of hearing aid of one ear

92593 - Check of hearing aid of both ears

92594 - Assessment of hearing aid function for one ear

92595 - Assessment of hearing aid function for both ears

F0DZ1KZ - Monaural Hearing Aid Device Fitting using Audiovisual Equipment

F0DZ1LZ - Monaural Hearing Aid Device Fitting using Assistive Listening Equipment

F0DZ1ZZ - Monaural Hearing Aid Device Fitting

F0DZ2KZ - Binaural Hearing Aid Device Fitting using Audiovisual Equipment

F0DZ2LZ - Binaural Hearing Aid Device Fitting using Assistive Listening Equipment

F0DZ2ZZ - Binaural Hearing Aid Device Fitting

F0DZ05Z - Tinnitus Masker Device Fitting using Hearing Aid Selection / Fitting / Test Equipment

F0DZ11Z - Monaural Hearing Aid Device Fitting using Audiometer

F0DZ12Z - Monaural Hearing Aid Device Fitting using Sound Field / Booth

F0DZ15Z - Monaural Hearing Aid Device Fitting using Hearing Aid Selection / Fitting / Test Equipment

F0DZ21Z - Binaural Hearing Aid Device Fitting using Audiometer

F0DZ22Z - Binaural Hearing Aid Device Fitting using Sound Field / Booth

F0DZ25Z - Binaural Hearing Aid Device Fitting using Hearing Aid Selection / Fitting / Test Equipment

F0DZ55Z - Assistive Listening Device Device Fitting using Hearing Aid Selection / Fitting / Test Equipment

F14 - Physical Rehabilitation and Diagnostic Audiology, Diagnostic Audiology, Hearing Aid Assessment

F14Z2KZ - Monaural Hearing Aid Assessment using Audiovisual Equipment

F14Z2LZ - Monaural Hearing Aid Assessment using Assistive Listening Equipment

F14Z2PZ - Monaural Hearing Aid Assessment using Computer

F14Z2ZZ - Monaural Hearing Aid Assessment

F14Z3KZ - Binaural Hearing Aid Assessment using Audiovisual Equipment

F14Z3LZ - Binaural Hearing Aid Assessment using Assistive Listening Equipment

F14Z3PZ - Binaural Hearing Aid Assessment using Computer

F14Z3ZZ - Binaural Hearing Aid Assessment

F14Z05Z - Cochlear Implant Assessment using Hearing Aid Selection / Fitting / Test Equipment

F14Z6ZZ - Binaural Electroacoustic Hearing Aid Check Assessment

F14Z8ZZ - Monaural Electroacoustic Hearing Aid Check Assessment

F14Z15Z - Ear Canal Probe Microphone Assessment using Hearing Aid Selection / Fitting / Test Equipment

F14Z21Z - Monaural Hearing Aid Assessment using Audiometer

F14Z22Z - Monaural Hearing Aid Assessment using Sound Field / Booth

F14Z23Z - Monaural Hearing Aid Assessment using Tympanometer

F14Z24Z - Monaural Hearing Aid Assessment using Electroacoustic Immitance / Acoustic Reflex Equipment

F14Z25Z - Monaural Hearing Aid Assessment using Hearing Aid Selection / Fitting / Test Equipment

F14Z31Z - Binaural Hearing Aid Assessment using Audiometer

F14Z32Z - Binaural Hearing Aid Assessment using Sound Field / Booth

F14Z33Z - Binaural Hearing Aid Assessment using Tympanometer

F14Z34Z - Binaural Hearing Aid Assessment using Electroacoustic Immitance / Acoustic Reflex Equipment

F14Z35Z - Binaural Hearing Aid Assessment using Hearing Aid Selection / Fitting / Test Equipment

F14Z55Z - Sensory Aids Assessment using Hearing Aid Selection / Fitting / Test Equipment

F14Z65Z - Binaural Electroacoustic Hearing Aid Check Assessment using Hearing Aid Selection / Fitting / Test Equipment

F14Z85Z - Monaural Electroacoustic Hearing Aid Check Assessment using Hearing Aid Selection / Fitting / Test Equipment

F15Z75Z - Tinnitus Masker Assessment using Hearing Aid Selection / Fitting / Test Equipment

Level 3: V5030-V5060 - Monaural Hearing Aid

Level 3: V5120-V5267 - Hearing Services - Hearing Aids

S0618 - Audiometry for hearing aid evaluation to determine the level and degree of hearing loss

S2230 - Implantation of magnetic component of semi-implantable hearing device on ossicles in middle ear

V532 - Fitting and adjustment of hearing aid

V5010 - Assessment for hearing aid

V5011 - Fitting/orientation/checking of hearing aid

V5014 - Repair/modification of a hearing aid

V5030 - Hearing aid, monaural, body worn, air conduction

V5040 - Hearing aid, monaural, body worn, bone conduction

V5050 - Hearing aid, monaural, in the ear

V5060 - Hearing aid, monaural, behind the ear

V5090 - Dispensing fee, unspecified hearing aid

V5100 - Hearing aid, bilateral, body worn

V5170 - Hearing aid, cros, in the ear

V5171 - Hearing aid, contralateral routing device, monaural, in the ear (ite)

V5171 - Hearing aid, contralateral routing device, monaural, in the ear (ite)

V5172 - Hearing aid, contralateral routing device, monaural, in the canal (itc)

V5180 - Hearing aid, cros, behind the ear

V5181 - Hearing aid, contralateral routing device, monaural, behind the ear (bte)

V5190 - Hearing aid, contralateral routing, monaural, glasses

V5210 - Hearing aid, bicros, in the ear

V5211 - Hearing aid, contralateral routing system, binaural, ite/ite

V5212 - Hearing aid, contralateral routing system, binaural, ite/itc

V5213 - Hearing aid, contralateral routing system, binaural, ite/bte

V5214 - Hearing aid, contralateral routing system, binaural, itc/itc

V5215 - Hearing aid, contralateral routing system, binaural, itc/bte

V5220 - Hearing aid, bicros, behind the ear

V5221 - Hearing aid, contralateral routing system, binaural, bte/bte

V5230 - Hearing aid, contralateral routing system, binaural, glasses

V5241 - Dispensing fee, monaural hearing aid, any type

V5242 - Hearing aid, analog, monaural, cic (completely in the ear canal)

V5243 - Hearing aid, analog, monaural, itc (in the canal)

V5244 - Hearing aid, digitally programmable analog, monaural, cic

V5245 - Hearing aid, digitally programmable, analog, monaural, itc

V5246 - Hearing aid, digitally programmable analog, monaural, ite (in the ear)

V5247 - Hearing aid, digitally programmable analog, monaural, bte (behind the ear)

V5248 - Hearing aid, analog, binaural, cic

V5249 - Hearing aid, analog, binaural, itc

V5250 - Hearing aid, digitally programmable analog, binaural, cic

V5251 - Hearing aid, digitally programmable analog, binaural, itc

V5252 - Hearing aid, digitally programmable, binaural, ite

V5253 - Hearing aid, digitally programmable, binaural, bte

V5254 - Hearing aid, digital, monaural, cic

V5255 - Hearing aid, digital, monaural, itc

V5256 - Hearing aid, digital, monaural, ite

V5257 - Hearing aid, digital, monaural, bte

V5258 - Hearing aid, digital, binaural, cic

V5259 - Hearing aid, digital, binaural, itc

V5260 - Hearing aid, digital, binaural, ite

V5261 - Hearing aid, digital, binaural, bte

V5262 - Hearing aid, disposable, any type, monaural

V5263 - Hearing aid, disposable, any type, binaural

V5267 - Hearing aid or assistive listening device/supplies/accessories, not otherwise specified

V5298 - Hearing aid, not otherwise classified

V5336 - Repair/modification of augmentative communicative system or device (excludes adaptive hearing aid)

Z461 - Encounter for fitting and adjustment of hearing aid

## PROC. - Hospitalization

99219 - Hospital observation care, typically 50 minutes

99223 - Initial hospital inpatient care, typically 70 minutes per day

99232 - Subsequent hospital inpatient care, typically 25 minutes per day

99283 - Emergency department visit, moderately severe problem

99284 - Emergency department visit, problem of high severity

99285 - Emergency department visit, problem with significant threat to life or function

99291 - Critical care delivery critically ill or injured patient, first 30-74 minutes

99472 - Subsequent inpatient hospital critical care of infant or young child, 29 days through 24 months of age, per day

G0378 - Hospital observation service, per hour

## PROC. - IPV

E0481 - Intrapulmonary percussive ventilation system and related accessories

## PROC. - Lung Function Measurement

82803 - Blood gases measurement

94010 - Measurement and graphic recording of total and timed exhaled air capacity

94060 - Measurement and graphic recording of the amount and speed of breathed air, before and following medication administration

94070 - Multiple measurements and graphic recordings of the amount and speed of breathed air, before and following medication administration

94150 - Measurement of largest amount of air exhaled from lungs

94375 - Respiratory diagnostic testing (flow volume loop)

94726 - Determination of lung volumes using plethysmography

94729 - Measurement of lung diffusing capacity

94760 - Measurement of oxygen saturation in blood using ear or finger device

## PROC. - Lung Transplant

0BYC0Z0 - Transplantation of Right Upper Lung Lobe, Allogeneic, Open Approach

0BYC0Z1 - Transplantation of Right Upper Lung Lobe, Syngeneic, Open Approach

0BYC0Z2 - Transplantation of Right Upper Lung Lobe, Zooplastic, Open Approach

0BYD0Z0 - Transplantation of Right Middle Lung Lobe, Allogeneic, Open Approach

0BYD0Z1 - Transplantation of Right Middle Lung Lobe, Syngeneic, Open Approach

0BYD0Z2 - Transplantation of Right Middle Lung Lobe, Zooplastic, Open Approach

0BYF0Z0 - Transplantation of Right Lower Lung Lobe, Allogeneic, Open Approach

0BYF0Z1 - Transplantation of Right Lower Lung Lobe, Syngeneic, Open Approach

0BYF0Z2 - Transplantation of Right Lower Lung Lobe, Zooplastic, Open Approach

0BYG0Z0 - Transplantation of Left Upper Lung Lobe, Allogeneic, Open Approach

0BYG0Z1 - Transplantation of Left Upper Lung Lobe, Syngeneic, Open Approach

0BYG0Z2 - Transplantation of Left Upper Lung Lobe, Zooplastic, Open Approach

0BYH0Z0 - Transplantation of Lung Lingula, Allogeneic, Open Approach

0BYH0Z1 - Transplantation of Lung Lingula, Syngeneic, Open Approach

0BYH0Z2 - Transplantation of Lung Lingula, Zooplastic, Open Approach

0BYJ0Z0 - Transplantation of Left Lower Lung Lobe, Allogeneic, Open Approach

0BYJ0Z1 - Transplantation of Left Lower Lung Lobe, Syngeneic, Open Approach

0BYJ0Z2 - Transplantation of Left Lower Lung Lobe, Zooplastic, Open Approach

0BYK0Z0 - Transplantation of Right Lung, Allogeneic, Open Approach

0BYK0Z1 - Transplantation of Right Lung, Syngeneic, Open Approach

0BYK0Z2 - Transplantation of Right Lung, Zooplastic, Open Approach

0BYL0Z0 - Transplantation of Left Lung, Allogeneic, Open Approach

0BYL0Z1 - Transplantation of Left Lung, Syngeneic, Open Approach

0BYL0Z2 - Transplantation of Left Lung, Zooplastic, Open Approach

0BYM0Z0 - Transplantation of Bilateral Lungs, Allogeneic, Open Approach

0BYM0Z1 - Transplantation of Bilateral Lungs, Syngeneic, Open Approach

0BYM0Z2 - Transplantation of Bilateral Lungs, Zooplastic, Open Approach

32851 - Transplant of lung

32852 - Transplant of lung on heart-lung machine

32853 - Transplant of both lungs

32854 - Transplant of both lungs on heart-lung machine

33935 - Transplantation of donor heart and lung

S2152 - Solid organ(s), complete or segmental, single organ or combination of organs; deceased or living donor(s), procurement, transplantation, and related complications; including: drugs; supplies; hospitalization with outpatient follow-up; medical/surgical, diagnostic, emergency, and rehabilitative services, and the number of days of pre- and post-transplant care in the global definition

## PROC. - Mastoidectomy

202 - Incision of mastoid and middle ear

204 - Mastoidectomy

2021 - Incision of mastoid

2041 - Simple mastoidectomy

2042 - Radical mastoidectomy

2049 - Other mastoidectomy

2092 - Revision of mastoidectomy

69220 - Removal of skin debris and drainage of mastoid cavity, simple

69222 - Removal of skin debris and drainage of mastoid cavity, complex

69501 - Incision of mastoid bone

69502 - Removal of mastoid bone

69505 - Removal of mastoid bone including removal of growth of middle ear

69511 - Removal of mastoid bone including removal of growth and bone of middle ear

69530 - Removal of portion of temporal bone including removal of mastoid bone

69552 - Removal of growth of external ear through mastoid bone

69601 - Revision of previous mastoid surgery with removal of remaining mastoid bone

69602 - Revision of previous mastoid surgery, modified radical procedure

69603 - Revision of previous mastoid surgery, radical procedure

69604 - Revision of previous mastoid surgery and ear drum

69605 - Revision of previous mastoid surgery

69635 - Repair of eardrum and ear canal with incision of mastoid bone

69636 - Repair of eardrum, ear canal, and bones with incision of mastoid bone

69637 - Repair of eardrum, ear canal, and bones with insertion of prosthesis with opening of mastoid

69641 - Repair of eardrum and ear canal with removal of mastoid bone, complex

69642 - Repair of eardrum, ear canal and bones with removal of mastoid bone, simple

69643 - Repair of eardrum and ear canal with removal of mastoid bone, simple

69644 - Repair of eardrum, ear canal and bones with removal of mastoid bone, with intact canal wall

69645 - Repair of eardrum and ear canal with removal of mastoid bone, extensive or radical

69646 - Repair of eardrum, ear canal and bones with removal of mastoid bone, extensive or radical

69715 - Removal of mastoid bone with implantation of cochlear stimulating system, accessed through the skin

69718 - Removal of mastoid bone with removal and replacement (accessed through the skin) of cochlear stimulating system

69910 - Removal of inner ear canal and removal of mastoid bone

## PROC. - Myringotomy Tubes

2001 - Myringotomy With Insertion Of Tube

69436 - Incision of eardrum with insertion of eardrum tube under general anesthesia

Z4582 - Encounter For Adjustment Or Removal Of Myringotomy Device (Stent) (Tube)

Z9622 - Myringotomy Tube(S) Status

## PROC. - Nasal Biopsy

30100 - Biopsy of lining of nose

31237 - Biopsy or removal of nasal polyp or tissue using an endoscope

## PROC. - Nitric Oxide Measurement

95012 - Measurement of inhaled nitric oxide gas

## PROC. - Oscillatory positive expiratory pressure

E0484 - Oscillatory positive expiratory pressure device, non-electric, any type, each

## PROC. - Sinus Surgery

22 - Operations on nasal sinuses

0121 - Incision and drainage of cranial sinus

00160 - Anesthesia for procedure on nose and sinus

00162 - Anesthesia for surgery of nose and sinus

00164 - Anesthesia for soft tissue biopsy on nose and sinus

220 - Aspiration and lavage of nasal sinus

221 - Diagnostic procedures on nasal sinus

224 - Frontal sinusotomy and sinusectomy

225 - Other nasal sinusotomy

226 - Other nasal sinusectomy

227 - Repair of nasal sinus

229 - Other operations on nasal sinuses

0406T - Examination of nasal passage and sinus using an endoscope with placement of implant

0407T - Examination of nasal passage and sinus using an endoscope with placement of implant, biopsy and removal of polyps

1821 - Excision of preauricular sinus

2200 - Aspiration and lavage of nasal sinus, not otherwise specified

2201 - Puncture of nasal sinus for aspiration or lavage

2202 - Aspiration or lavage of nasal sinus through natural ostium

2211 - Closed [endoscopic] [needle] biopsy of nasal sinus

2212 - Open biopsy of nasal sinus

2219 - Other diagnostic procedures on nasal sinuses

2241 - Frontal sinusotomy

2242 - Frontal sinusectomy

2253 - Incision of multiple nasal sinuses

2261 - Excision of lesion of maxillary sinus with Caldwell-Luc approach

2262 - Excision of lesion of maxillary sinus with other approach

2271 - Closure of nasal sinus fistula

2279 - Other repair of nasal sinus

8603 - Incision of pilonidal sinus or cyst

8621 - Excision of pilonidal cyst or sinus

8935 - Transillumination of nasal sinuses

21139 - Repair of frontal sinus through forehead

21343 - Open treatment of frontal sinus fracture

21344 - Open treatment of depressed frontal sinus fracture

30210 - Irrigation and drainage of sinus

30580 - Repair of abnormal drainage tract between two nasal sinuses

31000 - Irrigation of nasal sinus (maxillary)

31002 - Irrigation of nasal sinus (sphenoid)

31020 - Incision of nasal (maxillary) sinus through the nose

31030 - Create a window into the nasal (maxillary) sinus

31032 - Removal of nasal sinus growths

31040 - Incision through sinus at cheek bone to reach nerves and blood vessels

31050 - Incision of nasal (sphenoid) sinus

31070 - Incision of nasal (frontal) sinus

31075 - Incision of nasal sinus of one side of face

31080 - Insertion of material to stop growth of nasal sinus lining without a bone flap done through an incision below the eyebrow

31081 - Insertion of material to stop growth of nasal sinus lining without a bone flap done through an incision through the forehead

31084 - Insertion of material to stop growth of nasal sinus lining with a bone flap done through an incision below the eyebrow

31085 - Insertion of material to stop growth of nasal sinus lining with a bone flap done through an incision through the forehead

31086 - Incision under the eyebrow to drain the nasal (frontal) sinus with placement of bone graft

31087 - Incision through the forehead to drain the nasal (frontal) sinus with placement of bone graft

31090 - Removal of diseased tissue or growths in multiple nasal sinuses on one side of face

31200 - Partial removal of nasal sinus

31201 - Removal of nasal sinus from within the nose passage

31205 - Removal of nasal sinus from outside the nose passage

31225 - Removal of nasal sinus

31230 - Removal of nasal sinus and eye bone

31233 - Examination of nasal passage and sinus above teeth (maxillary sinus) using endoscope

31235 - Examination of nasal passage and sinus above eyes (sphenoid sinus) using endoscope

31253 - Complete examination of nose and sinuses using an endoscope

31254 - Partial removal of nasal sinus using an endoscope

31255 - Complete removal of nasal sinus using an endoscope

31256 - Incision of nasal (maxillary) sinus using an endoscope

31257 - Complete examination of nose and sinuses and removal of nasal sinus using an endoscope

31259 - Removal of tissue from sphenoid sinus using an endoscope

31267 - Removal of nasal sinus tissue using an endoscope, maxillary sinus

31276 - Exploration of nasal sinus using an endoscope

31287 - Incision of nasal (sphenoid) sinus using an endoscope

31288 - Removal of nasal sinus tissue using an endoscope, sphenoid sinus

31290 - Repair of leak of brain and spinal fluid from sinus behind bridge of nose using endoscope

31291 - Repair of leak of brain and spinal fluid from sinus behind eyes using endoscope

31295 - Dilation of maxillary sinus in the nose using an endoscope

31296 - Dilation of frontal sinus in the nose using an endoscope

31297 - Dilation of sphenoid sinus in the nose using an endoscope

31298 - Dilation of sphenoid and frontal sinus in the nose using an endoscope

31299 - Accessory sinus procedure

42260 - Repair of abnormal connection from nasal sinus to skin surface

61580 - Removal of nasal sinuses to approach brain lesion without the removal of the maxilla or eyeball

61581 - Removal of nasal sinuses to approach brain lesion with the removal of the maxilla or eyeball

61598 - Removal of skull to approach lesion or defect at skull base with tying of sinus

C9771 - Nasal/sinus endoscopy, cryoablation nasal tissue(s) and/or nerve(s), unilateral or bilateral

G2097 - Episodes where the patient had a competing diagnosis on or within three days after the episode date (e.g., intestinal infection, pertussis, bacterial infection, lyme disease, otitis media, acute sinusitis, chronic sinusitis, infection of the adenoids, prostatitis, cellulitis, mastoiditis, or bone infections, acute lymphadenitis, impetigo, skin staph infections, pneumonia/gonococcal infections, venereal disease (syphilis, chlamydia, inflammatory diseases [female reproductive organs]), infections of the kidney, cystitis or uti)

G9350 - Ct scan of the paranasal sinuses not ordered at the time of diagnosis or received within 28 days after date of diagnosis

G9354 - One ct scan or no ct scan of the paranasal sinuses ordered within 90 days after the date of diagnosis

S2342 - Nasal endoscopy for post-operative debridement following functional endoscopic sinus surgery, nasal and/or sinus cavity(s), unilateral or bilateral

S9024 - Paranasal sinus ultrasound

## PROC. - Detection of Micro-organsms

31624 - Irrigation and suction of lung airways to obtain cells using an endoscope

86140 - Measurement C-reactive protein for detection of infection or inflammation

86317 - Detection of infectious agent antibody, quantitative

87015 - Concentration of specimen for infectious agents

87040 - Bacterial blood culture

87070 - Bacterial culture, any other source except urine, blood or stool, aerobic ,

87071 - Bacterial culture and colony count

87075 - Bacterial culture, any source, except blood, anaerobic

87077 - Bacterial culture for aerobic isolates ,

87081 - Screening test for pathogenic organisms

87086 - Bacterial colony count, urine

87116 - Culture for acid-fast bacilli ,

87205 - Special Gram or Giemsa stain for microorganism ,

87206 - Special fluorescent and/or acid fast stain for microorganism

87299 - Detection test by immunofluorescent technique for organism

87486 - Detection test by nucleic acid for Chlamydia pneumoniae, amplified probe technique

87556 - Detection test by nucleic acid for Mycobacteria tuberculosis (TB bacteria), amplified probe technique

87581 - Detection test by nucleic acid for Mycoplasma pneumoniae (bacteria), amplified probe technique

87632 - Detection test by nucleic acid for multiple types of respiratory virus, multiple types or subtypes, 6-11 targets

87633 - Detection test by nucleic acid for multiple types of respiratory virus, multiple types or subtypes, 12-25 targets

87798 - Detection test by nucleic acid for organism, amplified probe technique

87804 - Detection test by immunoassay for influenza virus

87880 - Strep test by immunoassay for Streptococcus

88305 - Pathology examination of tissue using a microscope, intermediate complexity

88312 - Special stained specimen slides to identify organisms including interpretation and report

U0003 - Infectious agent detection by nucleic acid (dna or rna); severe acute respiratory syndrome coronavirus 2 (sars-cov-2) (coronavirus disease [covid-19]), amplified probe technique, making use of high throughput technologies as described by cms-2020-01-r

U0005 - Infectious agent detection by nucleic acid (dna or rna); severe acute respiratory syndrome coronavirus 2 (sars-cov-2) (coronavirus disease [covid-19]), amplified probe technique, cdc or non-cdc, making use of high throughput technologies, completed within 2 calendar days from date of specimen collection (list separately in addition to either hcpcs code u0003 or u0004) as described by cms-2020-01-r2

## PROC. - Sputum Induction

89220 - Sputum Specimen Collection

94640 - Respiratory inhaled pressure or nonpressure treatment to relieve airway obstruction or for sputum specimen

## PROC. - Steroid Injection

J1100 - Injection, dexamethasone sodium phosphate, 1 mg

J2704 - Injection, propofol, 10 mg

## PROC. - Sweat Chloride Test

82438 - Chloride level

89230 - Sweat collection

## PROC. - Tonsil surgery

0C5P0ZZ - Destruction of Tonsils, Open Approach

0C5P3ZZ - Destruction of Tonsils, Percutaneous Approach

0C5PXZZ - Destruction of Tonsils, External Approach

0C9P00Z - Drainage of Tonsils with Drainage Device, Open Approach

0C9P0ZX - Drainage of Tonsils, Open Approach, Diagnostic

0C9P0ZZ - Drainage of Tonsils, Open Approach

0C9P3ZX - Drainage of Tonsils, Percutaneous Approach, Diagnostic

0C9P3ZZ - Drainage of Tonsils, Percutaneous Approach

0C9P30Z - Drainage of Tonsils with Drainage Device, Percutaneous Approach

0C9PX0Z - Drainage of Tonsils with Drainage Device, External Approach

0C9PXZX - Drainage of Tonsils, External Approach, Diagnostic

0C9PXZZ - Drainage of Tonsils, External Approach

0CBP0ZX - Excision of Tonsils, Open Approach, Diagnostic

0CBP0ZZ - Excision of Tonsils, Open Approach

0CBP3ZX - Excision of Tonsils, Percutaneous Approach, Diagnostic

0CBP3ZZ - Excision of Tonsils, Percutaneous Approach

0CBPXZX - Excision of Tonsils, External Approach, Diagnostic

0CBPXZZ - Excision of Tonsils, External Approach

0CCP0ZZ - Extirpation of Matter from Tonsils, Open Approach

0CCP3ZZ - Extirpation of Matter from Tonsils, Percutaneous Approach

0CCPXZZ - Extirpation of Matter from Tonsils, External Approach

0CNP0ZZ - Release Tonsils, Open Approach

0CNP3ZZ - Release Tonsils, Percutaneous Approach

0CNPXZZ - Release Tonsils, External Approach

0CQP0ZZ - Repair Tonsils, Open Approach

0CQP3ZZ - Repair Tonsils, Percutaneous Approach

0CQPXZZ - Repair Tonsils, External Approach

0CTP0ZZ - Resection of Tonsils, Open Approach

0CTPXZZ - Resection of Tonsils, External Approach

42700 - Drainage of tonsil abscess

42820 - Removal of tonsils and adenoid glands patient younger than age 12

42821 - Removal of tonsils and adenoid glands patient age 12 or over

42825 - Removal of tonsils patient younger than age 12

42826 - Removal of tonsils patient age 12 or over

42842 - Removal of tonsils, tissue, muscle, and bone, without closure

42844 - Removal of tonsils, tissue, muscle, and bone, closure with local flap

42845 - Removal of tonsils, tissue, muscle, and bone, closure with other flap

42860 - Removal of remaining tonsil tissue

## PROC. - Tympanoplasty

69633 - Repair of eardrum, ear canal, and bones with insertion of prosthesis, without mastoidectomy

69635 - Repair of eardrum and ear canal with incision of mastoid bone

69636 - Repair of eardrum, ear canal, and bones with incision of mastoid bone

69637 - Repair of eardrum, ear canal, and bones with insertion of prosthesis with opening of mastoid

69642 - Repair of eardrum, ear canal and bones with removal of mastoid bone, simple

69643 - Repair of eardrum and ear canal with removal of mastoid bone, simple

69644 - Repair of eardrum, ear canal and bones with removal of mastoid bone, with intact canal wall

69646 - Repair of eardrum, ear canal and bones with removal of mastoid bone, extensive or radical

# **DRUG CODES**

We enumerated all codes for diagnoses and procedures but had to use regular expression (regex) searches to specify drugs since the number of separate codes in prescriptions was too large. Note that these are standard python regular expressions converted to lowercase.

These were grouped into the following categories:

- DRUG - Anti-inflammatory (Non-Steroid)
- DRUG - Anti-inflammatory (Steroid)
- DRUG - Antibiotics
- DRUG - Antidepressant
- DRUG - Antifungals
- DRUG - Antihistamine
- DRUG - Bronchodilators
- DRUG - Decongestant
- DRUG - Expectorant
- DRUG - Inhaled Hypertonic Saline
- DRUG - Mucolytics
- DRUG - Prophylactic Azithromycin

## DRUG - Antibiotics

(inject|oral).*(claforan|duriflex|fortaz|keflex|rocephin|suprax|vantin)

(tobramycin|plazomicin|streptomycin|gentamicin|amikacin) .* injection

amikacin.*inhalation suspension

amikacin.*injectable solution

amoxicillin.*(oral|inject)

ampicillin.*(oral|inject)

augmentin.*(oral|inject)

azithromycin.*(oral|inject)

aztreonam .* (injection|inhalation solution)

carbenicillin

ceftazidime.*inject

ciprofloxacin .* (oral|injection)

ciprofloxacin .* otic suspension

clarithromycin.*(oral|inject)

clindamycin.*(oral|inject)

colistin.*solution

doxycycline.*oral

erythromycin.*(oral|inject)

gatifloxacin

gentamicin.*(irrigation|injectable)

imipenem

levofloxacin.*(oral|inject)

moxifloxacin.*(oral|inject)

mupirocin.*nasal

ofloxacin.*(ophthalmic),

otic suspension .*cortisporin

piperacillin.*inject

sulfamethoxazole.*trimethoprim.*inject.*

sulfamethoxazole.*trimethoprim.*oral

tetracycline.*oral

tobradex

tobramycin.*inhalation solution

tobramycin.*injectable solution

tobramycin.*podhaler

vancomycin.*inject

## DRUG - Antidepressant

amitriptyline.*oral

bupropion.*oral

desvenlafaxine.*oral

duloxetine.*oral

fluoxetine.*oral

mirtazapine.*oral

nortriptyline.*oral

paroxetine.*oral

## DRUG - Antifungals

amphotericin .* (oral|inject)

clotrimazole.*otic

fluconazole.*(oral|inject)

itraconazole.*(oral|inject)

ketoconazole .* (oral|otic)

voriconazole.*(oral|otic)

## DRUG - Antihistamine

azelastine.*nasal

brompheniramine.*oral

cetirizine.*oral

chlorpheniramine.*oral

clemastine.*oral

diphenhydramine.*oral

exofenadine.*oral

levocetirizine.*oral

loratadine.*oral

## DRUG - Anti-inflammatory (Steroid)

beclomethasone dipropionate .* inhaler

budesonide .* (inhalation|inhaler|nasal spray)

dexamethasone .* injection

fluticasone .* (inhalation|inhaler|nasal spray)

prednisolone .* oral

prednisone .* oral

## DRUG - Anti-inflammatory (Non-Steroid)

ibuprofen .* oral

montelukast .* (oral|chewable)

naproxen .* oral

## DRUG - Bronchodilators

aclidinium.*inhaler.

albuterol [48] mg extended release oral tablet

albuterol.*(inhaler|inhalation)

atrovent

formoterol

indacaterol

ipratropium.*(inhalation|inhaler)

levalbuterol

olodaerol

pirbuterol

salmeterol .* inhaler

salmeterol

terbutaline

theophylline.*oral (tablet|capsule)

tiotropium

umeclidinium

vilanterol

## DRUG - Decongestant

(oxymetazoline|neosynephrine|xylometazoline|phenylephrin).*nasal

nasal.*afrin

## DRUG - Expectorant

guaifenesin

## DRUG - Mucolytics

acetylcysteine (5|25)00 mg effervescent oral tablet

acetylcysteine [12]00 mg/ml inhalation solution

acetylcysteine [56]00 mg oral (tablet|capsule)

bronchitol

dornase alfa 1 mg/ml inhalation solution

sodium chloride (4|5|6.5|6.9|7.4|9|9.5|10.5|11.5|20|21|26|26.5|30) mg/ml nasal (spray|solution)

sodium chloride 100 mg/ml inhalation solution

sodium chloride 30 mg/ml inhalation solution

sodium chloride 35 mg/ml inhalation solution

sodium chloride 60 mg/ml inhalation solution

sodium chloride 70 mg/ml inhalation solution
